# Increased expression of ATP binding cassette transporter genes following exposure of *Haemonchus contortus* larvae to a high concentration of monepantel in vitro

**DOI:** 10.1186/s13071-016-1806-9

**Published:** 2016-09-29

**Authors:** Ali Raza, Neil H. Bagnall, Abdul Jabbar, Steven R. Kopp, Andrew C. Kotze

**Affiliations:** 1 CSIRO Agriculture and Food, Queensland Bioscience Precinct, 306 Carmody Rd., St. Lucia, QLD 4067 Australia; 2 School of Veterinary Science, University of Queensland, Gatton, QLD 4343 Australia; 3 Faculty of Veterinary and Agricultural Sciences, The University of Melbourne, Werribee, VIC 3030 Australia

**Keywords:** Monepantel, ABC transporters, Gene induction, *Haemonchus contortus*, Ivermectin tolerance

## Abstract

**Background:**

There is some evidence that ATP binding cassette (ABC) transporters play a role in resistance to anthelmintics, particularly against macrocyclic lactones. Some anthelmintics, including ivermectin (IVM), have been shown to induce transcription of multiple ABC transporters in nematodes; however, the effects of monepantel (MPL) on transcription of these transporter genes has not been studied.

**Methods:**

Larvae of two MPL-susceptible isolates of *Haemonchus contortus* were exposed to MPL at two concentrations (2.5 and 250 μg/ml) for periods of 3, 6 and 24 h. Transcription levels of sixteen ABC transporter genes were measured at the end of the incubation periods. The consequences of MPL exposure were examined by measuring rhodamine-123 efflux from the larvae, and their sensitivity to subsequent treatment with IVM or levamisole.

**Results:**

Multiple ABC transporter genes showed significantly higher transcription in both worm isolates following exposure to MPL at 250 μg/ml for 3, 6 or 24 h, particularly the P-glycoprotein (P-gp) genes *pgp-11*, *pgp-12* and *pgp-14*. Of these, only *pgp-11* maintained the elevated levels 24 h after the end of the drug exposure period. In contrast, there was only a single instance of low-level upregulation as a result of exposure to MPL at 2.5 μg/ml. Larvae exposed to MPL at 250 μg/ml showed an increased efflux of rhodamine-123 and a proportion of the larval population showed an ability to subsequently tolerate higher concentrations of IVM in migration assays. There was no increased tolerance to IVM following pre-exposure to MPL at 2.5 μg/ml.

**Conclusions:**

Exposure of *H. contortus* larvae to 250 μg/ml MPL results in increased transcription of multiple transporter genes and increased R-123 efflux. The subsequent ability of a proportion of the larvae to tolerate IVM suggests a protective role of ABC transporters across different chemical entities. However, these observations were only made at a concentration of MPL well above that experienced by parasitic life stages in vivo, and hence their significance remains unclear.

**Electronic supplementary material:**

The online version of this article (doi:10.1186/s13071-016-1806-9) contains supplementary material, which is available to authorized users.

## Background

Monepantel (MPL) (trade name Zolvix®) was the first new anthelmintic for livestock use for over 25 years when it appeared in 2009. It was first introduced in New Zealand for the control of gastrointestinal nematodes (GINs) and associated diseases in small ruminants, and later in Australia and the United Kingdom in 2010. It shows broad-spectrum anthelmintic activity, targeting larval and adult stages of the most important species of sheep GINs [1, 2]. The compound is an amino-acetonitrile derivative (AAD) that acts as a positive allosteric modulator of a nematode-specific clade of nicotinic acetylcholine receptor (nAChR) subunits. Genetic screening of *Caenorhabditis elegans* revealed nAChR subunit ACR-23 as a target for MPL action [2], while Rufener et al. [3] suggested that Hco-MPTL-1 and other nAChR subunits of the DEG-3 subfamily are the targets for MPL in *H. contortus*.

Resistance to MPL was first reported by Scott et al. [4] in New Zealand. The authors reported that MPL showed no efficacy in terms of egg count reduction in goats or reduction of worm burden in sheep, and was ineffective against at least two GINs (*Teladorsagia circumcincta* and *Trichostrongylus colubriformis*). There is also evidence of MPL resistance in *Teladorsagia* spp. and *T. colubriformis* on a goat farm in New South Wales, Australia [5]. Resistance to MPL has also been reported in *H. contortus* in Uruguay, where the authors reported poor efficacy against this nematode with 42 and 82 % faecal egg count reduction at two different sheep farms [6]. A more recent study has also reported *H. contortus* population resistant to MPL at a sheep farm in Netherlands [7]. Early work on MPL resistance in laboratory-selected isolates of *H. contortus* showed that the resistance was associated with a number of changes to the target site. These mutations resulted in the generation of various truncated versions of the target receptor that would be expected to be non-functional [3]. However, there is no information at present on whether such target site changes are involved in any of the cases of resistance reported from the field. We were interested in whether ABC transporters may interact with MPL in nematodes, as a number of anthelmintics are known to be substrates of these transporters, and increases in efflux activity are associated with anthelmintic resistance in some cases, particularly for macrocyclic lactones [8–10]. As AAD compounds are hydrophobic, they may also be substrates for the drug transport proteins [11].

There is a considerable evidence that exposure to anthelmintics results in increased transcription of some ABC transporter genes, both in vitro [11–14] and in vivo [15–17]. These studies have generally concluded that such a transcriptional response suggests a role for the transporters in the efflux of the transcription-inducing anthelmintic, that is, the anthelmintic is likely a substrate for the transporters. Therefore, as a first step in understanding the interaction of efflux pathways with MPL in nematodes, the present study aimed to explore the effects of MPL exposure on the transcription patterns of ABC transporters in third-stage larvae (L3) of two MPL-susceptible isolates of *H. contortus.* We further investigated the phenotypic effects of this drug exposure by (i) measuring the efflux of the fluorescent dye rhodamine-123 (R-123) from the L3 pre-exposed to MPL compared to controls, and (ii) examining whether MPL pre-exposure enabled the L3 to tolerate subsequent exposure to ivermectin (IVM) and levamisole (LEV) in larval migration assays.

## Methods

### Parasite material

Two isolates of *H. contortus* were used in the present study: (i) Kirby, a drug susceptible isolate recovered from the field at the University of New England, Kirby Research Farm in 1986 [18]; and (ii) Wallangra (WAL), a multi-drug resistant isolate collected from the New England region of New South Wales (NSW) in 2003; at the time of isolation, it was resistant to benzimidazole, closantel, LEV, IVM and moxidectin [19]. The isolate has been further selected over at least five generations with moxidectin (Cydectin®) and is now unaffected by the recommended dose of moxidectin.

Both isolates were recovered from the field before the introduction of MPL, and show equivalent sensitivity to this drug in larval development assays [20]. Infected animals were housed at the Commonwealth Scientific and Industrial Research Organisation (CSIRO) Agriculture and Food FD McMaster laboratory at Armidale, NSW. Faeces were collected from infected animals and sent to the CSIRO Agriculture laboratories at the Queensland Bioscience Precinct, Brisbane, QLD, in zip-lock bags. Third-stage larvae (L3) were harvested from faecal cultures held at 27 °C for 7 days. The L3 were stored at 15 °C, and used for experiments within 4 weeks.

### In vitro monepantel exposure

The commercially available drench product Zolvix® (Novartis Animal Health, Australia) (25 mg MPL/ml) was used as a source of MPL in this study. Multiple separate anthelmintic solutions were prepared by two-fold serial dilutions in dimethyl-sulfoxide (DMSO).

Groups of approximately 30,000 L3 of each isolate were exposed to two concentrations of MPL (2.5 μg/ml and 250 μg/ml) and DMSO (vehicle control) for a range of time periods (3, 6 and 24 h). The DMSO concentration was 1 % (v/v) across all samples. The MPL concentrations were chosen based on:

(i) 250 μg/ml: the highest concentration that L3 could be exposed to while maintaining an ability to migrate at equivalent levels to controls in subsequent migration assays, that is, the highest concentration that could be used without compromising the fitness of the larvae as measured by migration assays (migration at 250 μg/ml approximately 90 % of controls) (unpublished data); and
(ii) 2.5 μg/ml: as an approximation of the range of MPL concentrations of 2–4 μg/g measured by Lifschitz et al. [21] in the abomasal contents of sheep within the first 48 h following administration of Zolvix®.

The larvae were kept on a roller-mixer (BTR-5, Ratek, Boronia, Australia) for the entire duration of the drug-exposure period. Three separate experiments were performed. At the end of the incubation period, larvae were processed in two ways:

(i) For use in RNA extraction, larvae were centrifuged (3000× *g*, 1 min), and the pellets were washed with 1 ml water, centrifuged again (3000× *g*, 1 min), and the pelleted L3 were stored at -80 °C.
(ii) For use in R-123 and migration assays, larvae were centrifuged (3000× *g*, 1 min), and the pellets were washed with 1 ml water, centrifuged again (3000× *g*, 1 min), and the L3 were resuspended in water and used *immediately*.

### qPCR analysis

RNA extraction, cDNA synthesis and qPCR were performed as described by Raza et al. [13]. Briefly, total RNA was extracted from 30,000 L3 using the RNeasy mini kit (Qiagen, Hilden, Germany) as recommended by the manufacturer. Turbo-DNase (Ambion, Carlsbad, USA) was used to remove genomic DNA. cDNA was synthesized using DNase-treated RNA with superscript III reverse transcriptase (Invitrogen, Carlsbad, USA) according to the manufacturer’s instructions. The cDNA samples were diluted to a concentration of 4 ng/ml for downstream applications.

The primers used in this study for quantitative PCR of 11 *pgp* genes, two multidrug-resistance protein genes (*mrp-1* and *mrp-5*), two genes from the ABCF family(*abcf-1* and *abcf-2*) and one half transporter gene (*haf-6*) were as reported by Raza et al. [13]. Three housekeeping genes (GAPDH, actin and β-tubulin) were used as reference genes for the qPCR analysis (see Additional file 1: Table S1). The SYBR Green dye system (Applied Biosystems, Warrington, UK) was used in a Vii A7 thermocycler (Applied Biosystems, USA) under the following PCR cycling conditions: 50 °C for 2 min, 95 °C for 10 min (stage I), followed by 40 cycles of 95 °C for 15 s, 60 °C for 1 min (stage II) and a melt curve stage at 95 °C for 15 s, 60 °C for 1 min and 95 °C for 15 s (stage III). Three separate extractions for each treatment were examined, with each PCR run in quadruplicate. Reaction efficiencies, determined by standard curves, were in the range between 80 and 99 %. The homogeneity of the PCR products was ensured by (i) analysing the melt-curves in each run, (ii) visualizing a single band for each PCR product on 2 % electrophoresis gels, and (iii) cloning of the selected primer products into the vector PCR2.1 (Invitrogen, USA) followed by sequencing (Big-Dye terminator, V3.1; Applied Biosystems, USA) using M13 forward/reverse primers. Expression values for all genes in each sample were normalised to the housekeeping genes using REST 2009 (version v2.0.13) to determine the effects of MPL pre-exposure on the transcription of ABC transporters (drug exposure regimen described above) using DMSO treated samples as control for each isolate. The triplicate expression values were log_10_ transformed and analysed using repeated measures ANOVA with Fischer’s Least Significant Difference (LSD) *post-hoc* test (*P* < 0.05) to compare the transcription profiles of control and MPL-exposed samples in GraphPad Prism(version 6.01).

### Rhodamine (R-123) efflux assay

R-123 efflux was measured in order to determine the effects of MPL pre-exposure (2.5 μg/ml and 250 μg/ml) on the efflux activity of ABC transporters in Kirby and WAL L3stage larvae, using the protocols described earlier [13]. Briefly, approximately 20,000 L3 were exposed to MPL or DMSO for 3 and 6 h (as described above) and then placed into a solution of R-123 (2 ml of 1.5 μM) for 15 min in the dark at room temperature on a roller-mixer. The worms were centrifuged (3000× *g*, 1 min) and washed with 1 ml of distilled water. The pellet was resuspended in distilled water (1 ml) and placed on a roller for 60 min in the dark. Finally, the worms were centrifuged (3000× *g*, 1 min) and the supernatant was collected and stored in dark for 60 min before use. R-123 concentration in the supernatant was detected by measuring the specific fluorescence (λ for excitation = 495 nm and λ for emission = 525 nm) using a spectrophotometer (Spectra Max M3, Molecular Devices®, Sunnyvale, CA, USA). The concentration of R-123 in each experimental sample was calculated from a standard curve, determined using a range of R-123 concentrations. Each experiment consisted of duplicate incubations for each treatment. Three separate experiments were performed and data (*n* = 3 for each treatment) were log_10_-transformed and analysed using repeated measures ANOVA followed by Fischer’s LSD test (*P* < 0.05) (GraphPad Prism, version 6.01).

### Larval migration assay (LMA)

LMAs were used to measure the changes in tolerance of Kirby and WAL L3 to IVM and LEV following pre-exposure to MPL for 3 or 6 h. We were unable to assess whether MPL pre-exposure resulted in tolerance to MPL itself as this drug does not inhibit larval migration, even at high concentrations, and is therefore not suitable for use in LMAs [20]. The assay was performed following the procedure as reported by Raza et al. [13]. This method was based on that described earlier by Kotze et al. [22], except with the drug exposure and migration periods reduced from 24–48 h to 30 min each in order to allow for assessment of migration inhibition without providing any time for drug-induced gene expression changes to occur during the time course of the migration assay itself.

Briefly, MPL pre-treated (2.5 μg/ml and 250 μg/ml) or control (pre-exposure to 1 % DMSO only) larvae (3800 L3/ ml of water) were exposed to a range of IVM and LEV concentrations in 96-well microtiter plates for 30 min at 27 °C. Final concentrations for IVM and LEV ranged from 100-0.195 μg/ml and 12.5–0.024 μg/ml, respectively. After 30 min, the larvae were collected using a multi-tip pipette and placed into migration plates with 20 μm filters above receiver plate wells (Millipore, Bayswater, Australia). The larvae were allowed to migrate through the filters for a period of 30 min, before the filter plates were removed and the larvae in the receiver wells killed using Lugol’s iodine, and counted.

Migration assays consisted of triplicate assay wells at each IVM or LEV concentration. Three separate experiments were performed for each isolate. Data were analysed using non-linear regression in GraphPad Prism (version 6.01). IC_50_ values and 95 % confidence intervals were calculated based on the pooled data from each set of nine assays, and significant differences were determined by the overlap of 95 % confidence intervals.

The dose response to IVM in the short-term migration assays used in the present study showed the presence of a plateau in the response at the highest drug concentrations, as described previously by Raza et al. [13]. The percent migration remained at a constant level (above 0 %) over the highest 2 to 3 drug concentrations. Hence, for the analysis of the IVM dose-response data, we used a non-normalised model in GraphPad (‘top to bottom’), with a variable slope. The output of this analysis provided us with two parameters with which to compare populations: first, the percent migration at the dose-response plateau (that is, the percentage of the population unaffected by the highest concentrations of IVM in the assay), and secondly, the IC_50_ in the remaining proportion of the population that had shown a dose-response to the drug (for instance, if the plateau existed at a level of 30 % migration, then the IC_50_ value defined the response to the drug in the remaining 70 % of the worm population alone). In addition, to further determine the significant differences between the percentage of larval migration following exposure to DMSO and MPL, the percent migration in MPL-pre-exposed compared to DMSO-pre-exposed worms at each IVM concentration were compared using a *t*-test with Welch’s correction (*P* < 0.05) (GraphPad Prism).

## Results

### Transcriptional response of ABC transporters to MPL exposure in Kirby larvae

**Fig. 1:**
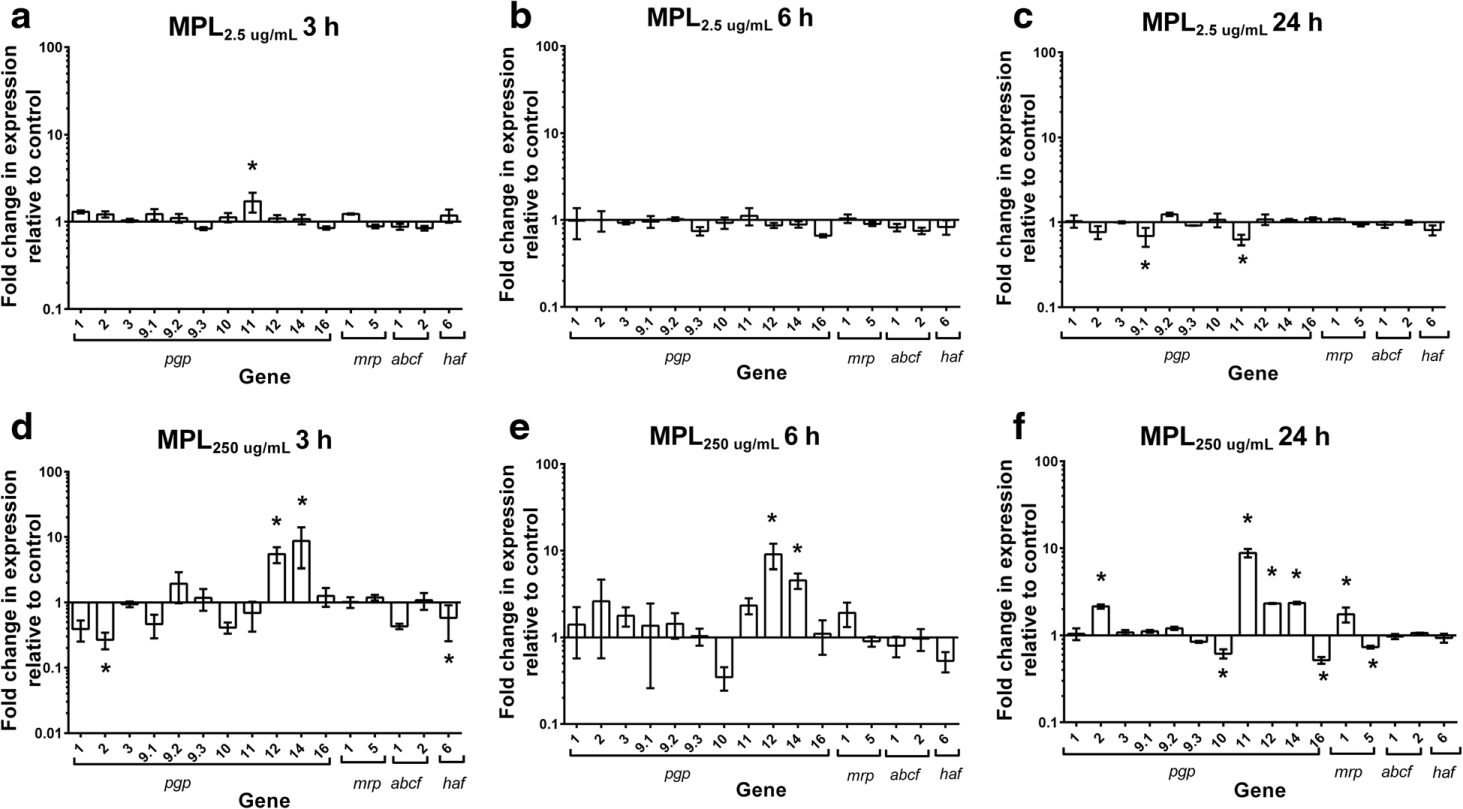
Effect of monepantel on transcription patterns of ABC transporter genes in *Haemonchus contortus* Kirby isolate. Larvae were exposed to MPL at two concentrations for a range of time periods: MPL_2.5μg/ml_ (**a** at 3 h, **b** at 6 h and **c** at 24 h) or MPL_250μg/ml_ (**d** at 3 h, **e** at 6 h and **f** at 24 h). Y-axis shows fold-change in gene expression levels in drug-treated larvae *vs* DMSO-treated controls. Data shown as mean ± standard error of the mean (SEM), *n* = 3 separate experiments each with four technical replicates. Significant differences in gene transcription levels are indicated by * (*P* < 0.05)

Figure 1 shows the transcription patterns of ABC transporters following exposure of Kirby L3 to MPL at 2.5 μg/ml (panels a, b and c) or 250 μg/ml (panels d, e and f) for 3, 6 and 24 h, relative to control larvae (exposed to DMSO only). The fold changes in expression levels in response to 250 μg/ml MPL (relative to DMSO-treated controls) are shown in Table 1. There were instances of increased and decreased expression of transporter genes across the various time points in MPL-treated L3 compared to controls, with more instances of increased rather than decreased expression levels, and with increases being generally of a greater magnitude (fold change) than the decreases (ANOVA: 2.5 μg/ml, 3 h *F*_(16,32)_ = 3.227, *P* = 0.0023, 6 h *F*_(16,32)_ = 1.070, *P* = 0.4191, 24 h *F*_(16,32)_ = 2.509, *P* = 0.0131; 250 μg/ml, 3 h *F*_(16,32)_ = 5.224, *P* < 0.0001, 6 h *F*_(16,32)_ = 3.037, *P* = 0.0036, 24 h *F*_(16,32)_ = 69.82, *P* < 0.0001). The transcriptional responses to MPL_250 μg/ml_ were greater than for MPL_2.5 μg/ml_, in terms of both the number of significant changes, and their magnitude. The only instance of significant gene upregulation following treatment with MPL_2.5 μg/ml_ was a 1.7-fold increase in transcription level of *pgp-11* (*P* = 0.002) at 3 h (Fig. 1a). There were no significant changes in transcription patterns of any of the transporter genes after 6 h exposure to MPL_2.5 μg/ml_ (Fig. 1b); whereas, after 24 h exposure, there was significant downregulation of *pgp-9.1* (1.5-fold; *P* = 0.01) and *pgp-11* (1.6-fold; *P* = 0.007) (Fig. 1c).

**Table 1:** Relative transcription levels of ABC transporter genes in the 250 μg/ml MPL-treated Kirby and WAL larvae compared to DMSO-treated controls. Significant (*P* < 0.05) increases or decreases in transcription in the MPL-treated larvae compared to the DMSO-treated larvae are indicated by * and #, respectively

Gene	Kirby			WAL		
	3 h	6 h	24 h	3 h	6 h	24 h
Pgp 1	0.39	1.4	1.0	2.5	0.77	0.98
Pgp 2	0.29^#^	2.6	2.2*	7.9*	2.1	2.2*
Pgp 3	0.94	1.8	1.1	2.7	0.68	1.2
Pgp 9.1	0.46	1.4	1.1	8.4*	1.2	1.0
Pgp 9.2	1.9	1.4	1.2	1.8	0.97	0.95
Pgp 9.3	1.2	1.0	0.84	1.0	1.4	0.72^#^
Pgp 10	0.41	0.35	0.62^#^	1.8	0.76	0.59^#^
Pgp 11	0.69	2.3	8.8*	11.7*	7.4*	6.9*
Pgp 12	5.4*	9.1*	2.3*	9.4*	8.2*	2.9*
Pgp 14	8.7*	4.6*	2.4*	12.2*	5.5*	2.6*
Pgp 16	1.3	1.1	0.52^#^	0.51	1.0	0.79^#^
MRP 1	1.01	1.9	1.8*	1.2	1.0	1.5*
MRP 5	1.2	0.9	0.73^#^	1.4	0.88	0.79^#^
ABCF 1	0.43	0.8	0.97	2.0	0.87	0.92
ABCF 2	1.1	0.97	1.1	2.1	1.0	1.0
HAF-6	0.58^#^	0.54	0.94	20.5*	0.85	1.0

# denotes decreased gene transcription following MPL exposure compared to DMSO-treated controls

* denotes increased gene transcription following MPL exposure compared to DMSO-treated controls

Following exposure to MPL_250 μg/ml_ for 3 and 6 h, *pgp-12* transcription was increased by 5.4-fold (*P* = 0.007) and 9.1-fold (*P* = 0.002), respectively, while *pgp-14* showed 8.7-fold (*P* = 0.002) and 4.6-fold (*P* = 0.03) increases, respectively (Fig. 1d, e) (Table 1). Exposure to MPL_250 μg/ml_ for 3 h resulted in significant downregulation of *pgp-2* and *haf-6* by 3.4-fold (*P* = 0.002) and 1.7-fold (*P* = 0.02), respectively. These decreases in transcription were short-lived, as, by 6 h, transcription levels for these two genes had returned to levels equivalent to DMSO-controls (Fig. 1d, e). Four P-gp genes and *mrp-1* were significantly upregulated (*P* < 0.0001) following 24 h exposure to MPL_250 μg/ml_; the increases were as follows: 8.8-fold for *pgp-11*, 2.4-fold for *pgp-14*, 2.3-fold for *pgp-12*, 2.2-fold for *pgp-2* and 1.8-fold for* mrp-1*. In addition, there were some instances of downregulation, with *pgp-10* (1.6-fold, *P* < 0.0001), *pgp-16* (2-fold, *P* < 0.0001) and *mrp-5* (1.4-fold, *P* = 0.009) showing significantly decreased transcriptions relative to DMSO controls (Fig. 1f) (Table 1). The most consistent changes in larval gene expression following exposure to MPL_250 μg/ml_ were the increased transcription of *pgp-12* and *pgp-14* at each of the three time points. There was a single instance of inconsistent changes in gene expression values between the two MPL concentrations: after 24 h exposure, *pgp-11* showed an increased expression level in response to MPL_250 μg/ml_ (8.8-fold), compared to a decrease in expression in response toMPL_2.5 μg/ml_ (1.6-fold) (compare Fig. 1c, f).

### Transcriptional response of ABC transporters to MPL exposure in WAL larvae

**Fig. 2:**
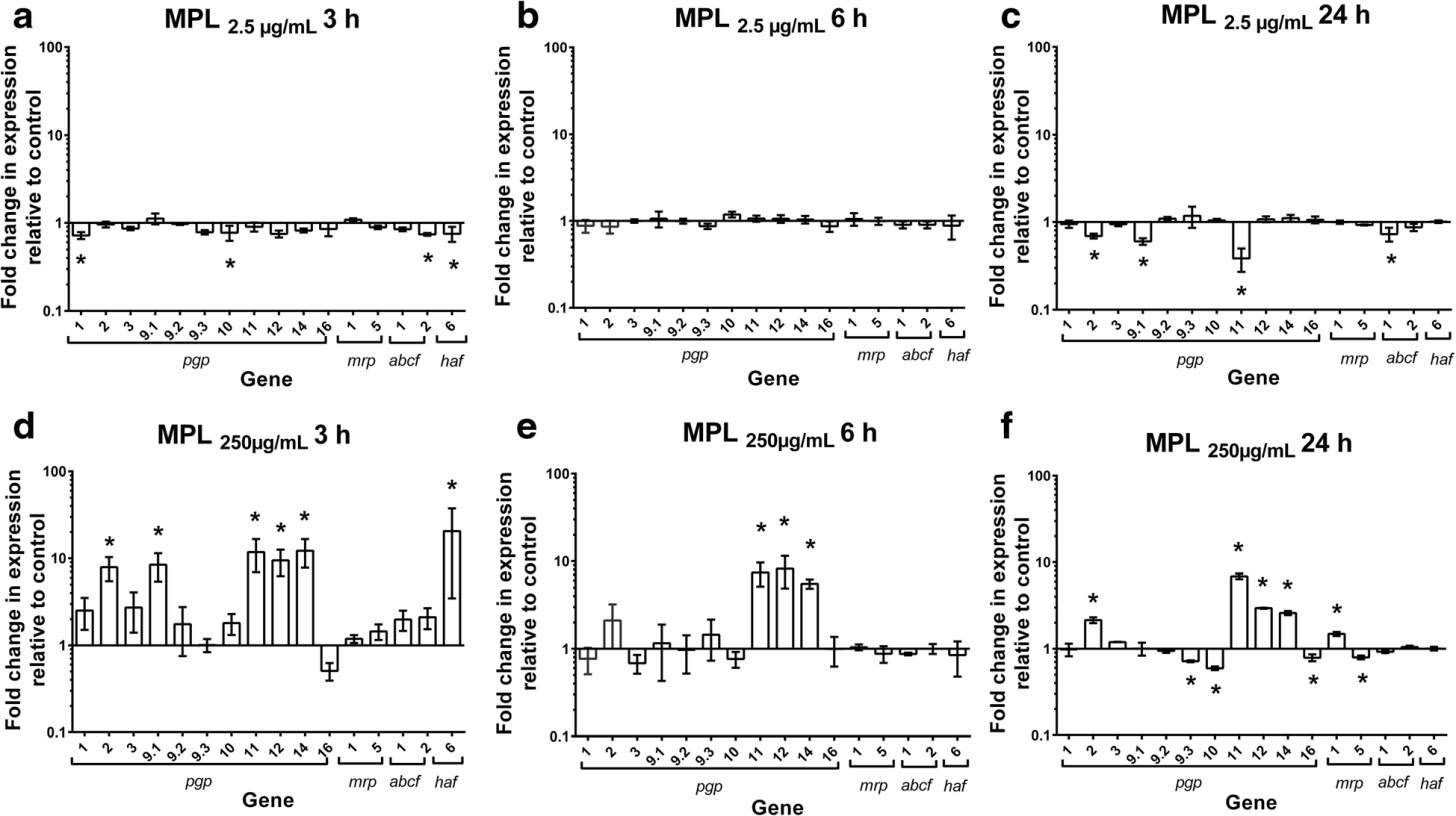
Effect of monepantel on transcription patterns of ABC transporter genes in *Haemonchus contortus* WAL isolate. Larvae were exposed to MPL at two concentrations for a range of time periods: MPL_2.5μg/ml_ (**a** at 3 h, **b** at 6 h and **c** at 24 h) or MPL_250μg/ml_ (**d** at 3 h, **e** at 6 h and **f** at 24 h). Y-axis shows fold-change in gene expression levels in drug-treated larvae *vs* DMSO-treated controls. Data shown as mean ± standard error of the mean (SEM), *n* = 3 separate experiments each with four technical replicates. Significant differences in gene transcription levels are indicated by * (*P* < 0.05)

The effects of MPL on transcription of ABC transporters in WAL larvae are shown in Fig. 2 and Table 1 (ANOVA: 2.5 μg/ml, 3 h *F*_(16,32)_ = 1.793, *P* = 0.0782, 6 h *F*_(16,32)_ = 1.057, *P* = 0.4305, 24 h *F*_(16,32)_ = 7.058, *P* < 0.0001; 250 μg/ml, 3 h *F*_(16,32)_ = 5.789, *P* < 0.0001, 6 h *F*_(16,32)_ = 4.831, *P* < 0.0001, 24 h *F*_(16,32)_ = 71.99, *P* < 0.0001). Exposure of WAL L3 to MPL_2.5 μg/ml_ did not result in significant upregulation of any of the transporter genes (Fig. 2a, c). On the other hand, there were a number of instances of significant downregulation at 3 and 24 h (Fig. 2a, c). The magnitude of these decreases at 3 h were as follows: 1.4-fold for *pgp-1* and *abcf-1* (*P* = 0.02; *P* = 0.04), and 1.3-fold for *pgp-10* and *haf-6* (*P* = 0.04; *P* = 0.03). These decreases in gene transcription were short lived as they had returned to levels equivalent to controls by 6 h exposure (Fig. 2b). Following 24 h MPL_2.5 μg/ml_ exposure, *pgp-2* (1.4-fold; *P* = 0.02), *pgp-9.1* (1.6-fold; *P* = 0.002), *pgp-11* (2.6-fold; *P* < 0.0001) and *abcf-1* (1.4-fold; *P* = 0.03) showed significantly decreased transcription relative to DMSO controls (Fig. 2c).

In contrast, exposure of WAL larvae to MPL_250 μg/ml_ for 3 h resulted in significantly increased transcription of five P-gp genes and the *haf*-6 (Fig. 2d) (Table 1). Of note here was the greater number of upregulated genes in WAL compared to Kirby isolate following 3 h exposure to MPL (compare Figs. 1d and 2d). The magnitudes of the increases for WAL were as follows: 7.9-fold for *pgp-2* (*P* = 0.002), 8.4-fold for *pgp-9.1* (*P* = 0.002), 11.7-fold for *pgp*-*11* (*P* = 0.0004), 9.4-fold for *pgp-12* (*P* = 0.001), 12.2-fold for *pgp-14* (*P* = 0.0002) and 20.5-fold for *haf-6* (*P* = 0.0007). There were no instances of significant downregulation at the 3 h and 6 h time points. The increased transcription of *pgp-2*, *pgp-9.1* and *haf-6* genes observed at 3 h was short lived as it had returned to DMSO control levels by the 6 h time point for these three genes (Fig. 2d, e) (Table 1). In contrast, the upregulation of *pgp-11*, *pgp-12* and *pgp-14* was maintained at the 6 h time point; *pgp-11 *7.4-fold (*P* = 0.0007), *pgp-12* 8.2-fold (*P* = 0.001), and *pgp-14* 5.5-fold (*P* = 0.002). These increases also occurred at the 24 h time point; *pgp-11* 6.9-fold (*P* < 0.0001), *pgp-12* 2.9-fold (*P* < 0.0001) and *pgp-14* 2.6-fold (*P* < 0.0001). For each of these genes, the magnitude of fold change in expression relative to controls decreased over the sequential time points from 3 to 24 h (for example from 12.2-fold to 2.6-fold for *pgp-14*). Following 24 h exposure to MPL, transcription of *pgp-9.3*, *pgp-10*, *pgp-16* and *mrp-5* were significantly downregulated at levels of 1.4-fold (*P* = 0.003), 1.7-fold (*P* < 0.0001), 1.3-fold (*P* = 0.02) and 1.3-fold (*P* = 0.03), respectively (Fig. 2f).

There were several instances of inconsistent changes in gene expression values between the two MPL concentrations, with expression reduced at the lower MPL concentration and increased at the higher concentration for *haf-6* at 3 h, as well as *pgp-2* and *pgp-11* at 24 h. As described above, this difference in response to the two MPL concentrations was also observed for Kirby with *pgp-11* at 24 h.

Overall, for WAL larvae, the most marked effects of MPL exposure were the significant upregulation of a number of transporter genes at the 3 h time point for MPL_250 μg/ml_, and the sustained nature of these increases for *pgp-11*, *pgp-12* and *pgp-14*, alongside the absence of any upregulation by MPL_2.5 μg/ml_. This pattern was very similar to that seen with Kirby larvae, with the exception of the unchanged expression for *pgp-11* at 3 h for the Kirby larvae (Table 1).

**Fig. 3:**
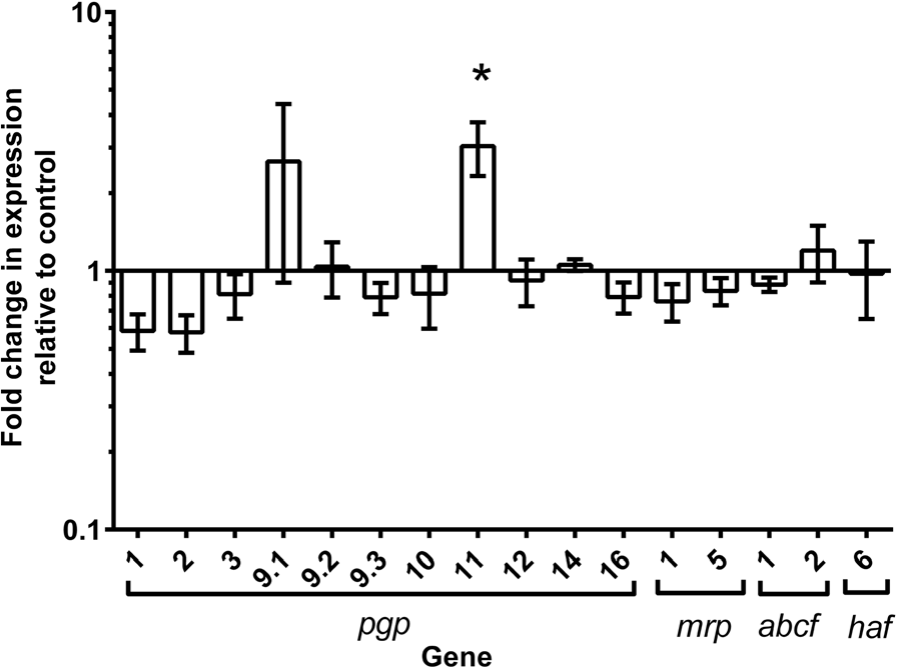
Stability of transporter gene transcription following 3 h pre-exposure of WAL larvae to monepantel. Larvae were exposed to MPL_250μg/ml_ for 3 h, rinsed in water and maintained for 24 h with no drug. Y-axis shows fold-change in gene expression levels in drug-treated *vs* DMSO-treated larvae. Data shown as mean ± standard error of the mean (SEM), *n* = 3 separate experiments each with four technical replicates. Significant differences in transcription levels are indicated by * (*P* < 0.05)

The stability of the observed transcriptional changes was examined by exposing WAL L3 to MPL_250 μg/ml_ for 3 h, rinsing the larvae in water, and then maintaining them in the absence of drug for a further 24 h before recovery for analysis of ABC transporter gene expression patterns. Figure 3 shows that of the gene expression changes measured in WAL L3 after 3 h exposure to MPL (from Fig. 2d), only the increased expression of *pgp-11* was maintained in these larvae after 24 h in the absence of any drug.

Over this period, the fold increase in this gene in MPL-treated larvae compared to controls decreased from 11.7-fold (*P* = 0.0004) to 3-fold (*P* = 0.004) (compare Figs. 2d and 3). There was some variability in the expression patterns of *pgp-9.1* (as indicated by the large SEM bars), however, statistical analysis across the three replicate experiments showed that the expression level of this gene was not significantly different to controls.

### Phenotypic characterization of the MPL-treated larvae

#### Rhodamine-123 efflux assay

**Fig. 4:**
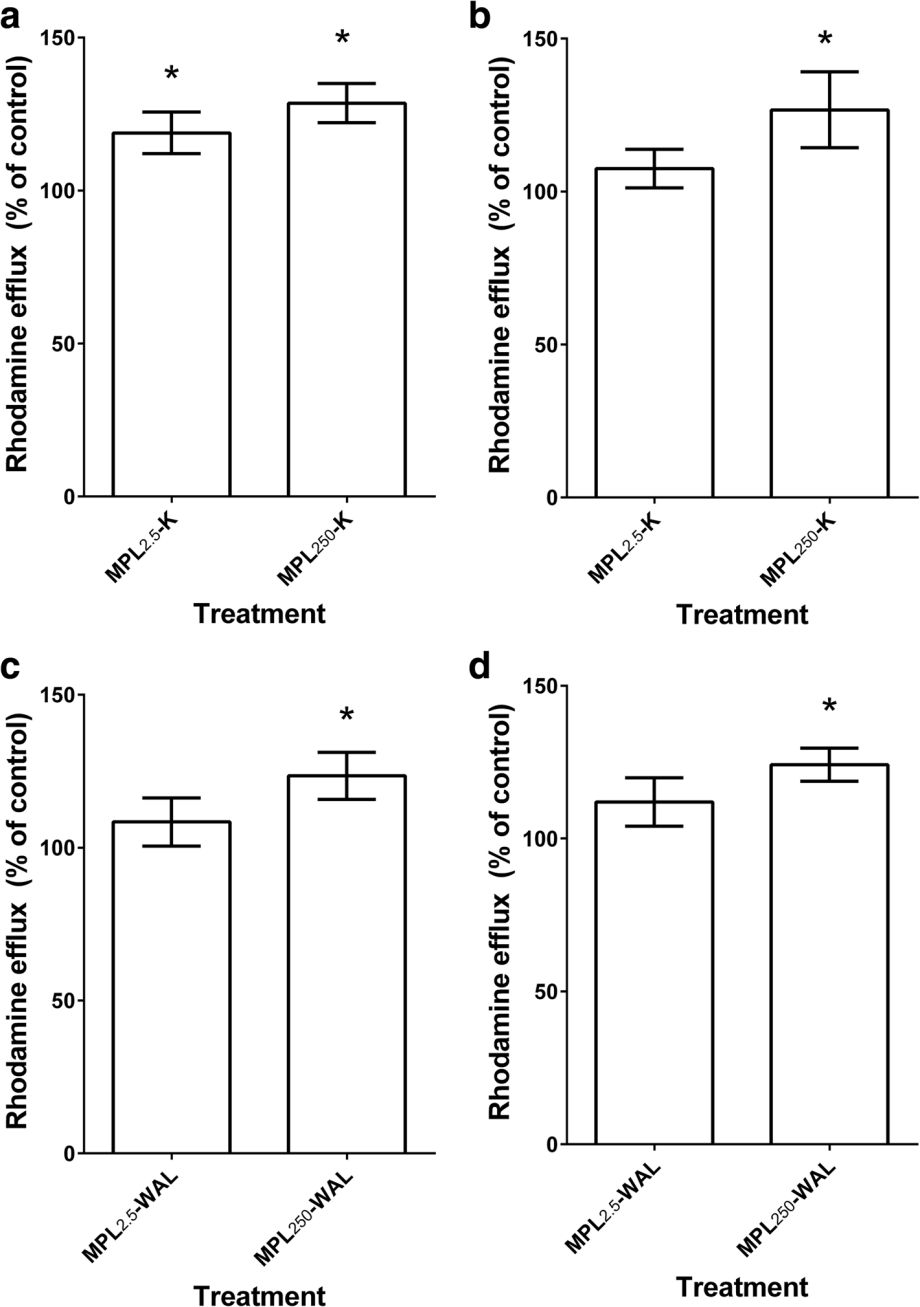
Rhodamine efflux from larvae following monepantel pre-exposure. R-123 efflux was measured after exposure of L3 stage larvae to MPL_250μg/ml_ or MPL_2.5μg/ml_, compared to DMSO-treated controls; Kirby isolate: **a** at 3 h and **b** at 6 h; WAL isolate: **c** at 3 h and **d** at 6 h. Significant increase in R-123 efflux compared to controls is indicated by * (*P* < 0.05). The columns represent mean ± standard error of the mean (SEM), *n* = 6 (pooled data from three separate experiments, each with assays in duplicate)

The functional consequences of exposure to MPL were evaluated by measuring the ability of MPL pre-treated Kirby and WAL L3 to efflux the fluorescent dye R-123 (Fig. 4) (ANOVA: Kirby 3 h *F*_(2,4)_ = 12.51, *P* = 0.0190, 6 h *F*_(2,4)_ = 6.004, *P* = 0.0624; WAL 3 h *F*_(2,4)_ = 4.148, *P* = 0.1058, 6 h *F*_(2,4)_ = 9.691, *P* = 0.0293). Exposure of Kirby L3 to MPL_2.5 μg/ml_ and MPL_250 μg/ml_ for 3 h resulted in increased R-123 efflux compared to controls (1.2-fold, *P* = 0.009, and 1.3-fold, *P* = 0.017, respectively) (Fig. 4a). By 6 h, increased efflux was still recorded for worms exposed to MPL_250 μg/ml_ (1.2-fold, *P* = 0.027), whereas the efflux had returned to control levels for the MPL_2.5 μg/ml_ treatment (Fig. 4b). For WAL larvae, increased efflux was observed for MPL_250 μg/ml_ at both time points (1.2-fold, *P* = 0.045 at 3 h, and 1.2-fold, *P* = 0.011 at 6 h), while efflux was unchanged for the MPL_2.5 μg/ml_ treatment at both time points (Fig. 4c, d).

The levels of R-123 efflux were equivalent in Kirby and WAL L3 in the absence of any drug exposure (DMSO-treated) at both 3 and 6 h (*P* = 0.24 and *P* = 0.12) (data not shown).

#### Larval migration assay

The functional consequences of changes in transcription of transporter genes following exposure to the two concentrations of MPL were further examined using migration assays. Following 3 h exposure to MPL_2.5 μg/ml_, there were no significant changes to the tolerance of both Kirby and WAL L3to IVM and LEV, both in terms of the IVM IC_50_ values and the percent migration at the dose-response plateau at the high IVM concentrations (data not shown). Given, this lack of effect of 3 h exposure to MPL_2.5 μg/ml_, alongside the absence of gene expression changes in 6 h MPL_2.5 μg/ml_-treated Kirby and WAL larvae (Figs. 1b, 2b), we did not measure the response of 6 h MPL_2.5 μg/ml_ pre-exposed larvae to IVM or LEV.

**Fig. 5:**
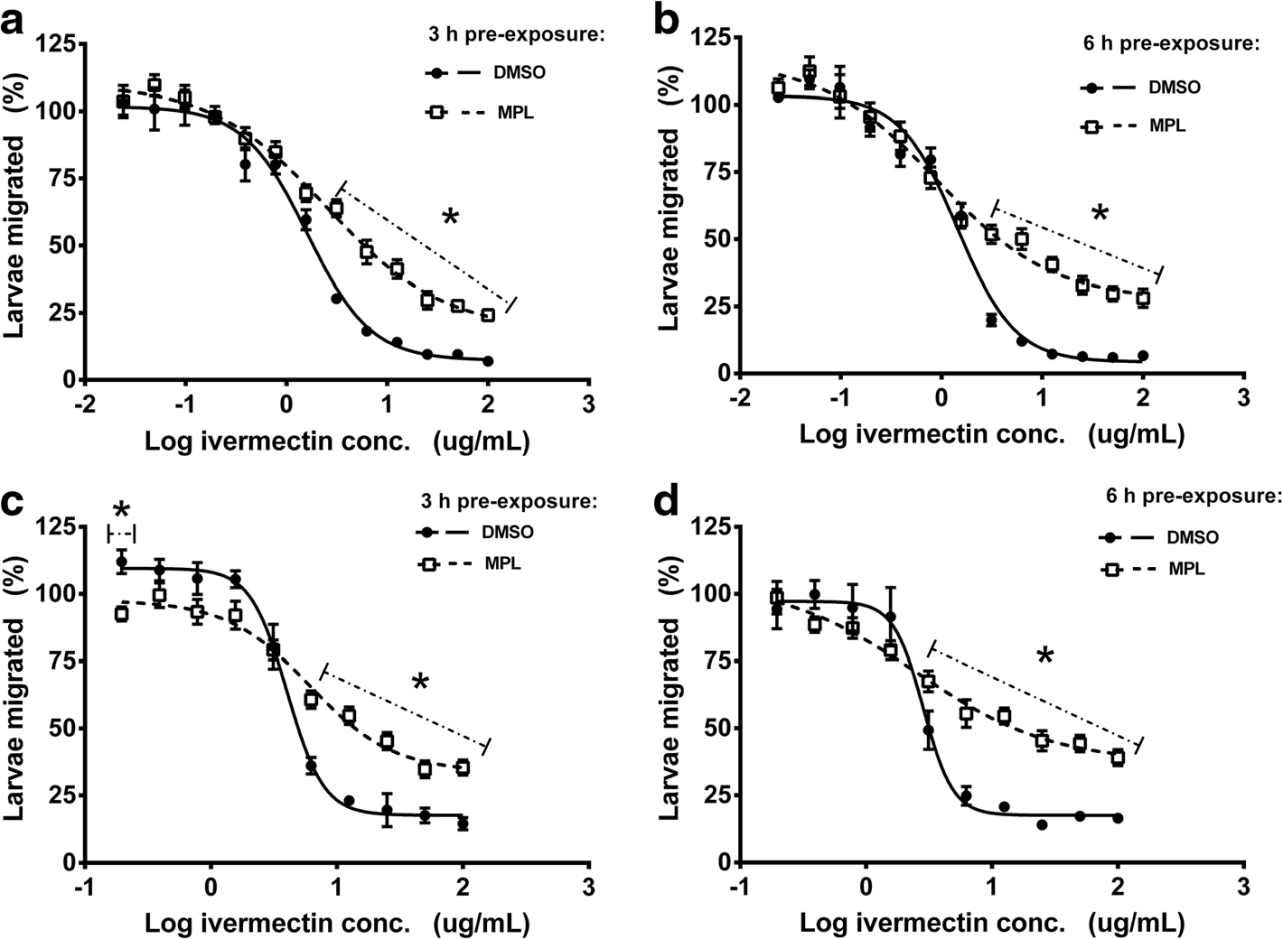
IVM sensitivity in Kirby and WAL L3 stage larvae following monepantel pre-exposure. Sensitivity to IVM was measured using migration assays following pre-exposure to MPL_250μg/ml_ in Kirby (**a** after 3 h pre-exposure and **b** after 6 h pre-exposure) and WAL (**c** after 3 h pre-exposure and **d** after 6 h pre-exposure) L3 stage larvae, compared to L3 pre-exposed to DMSO only. DMSO pre-exposure shown with solid lines and closed symbols, MPL pre-exposure shown with dashed lines and open symbols. The data points marked with * show significant differences (*P* < 0.05) in percent migration between MPL- and DMSO- pre-exposure groups (*t*-test). Each data point represents mean ± standard error of the mean (SEM), *n* = 9 (pooled data from three experiments, each with assays in triplicate)

In contrast to the 3 h MPL_2.5 μg/ml_ data, exposure to MPL_250 μg/ml_ for 3 or 6 h resulted in significant changes in the IVM dose response curves compared to larvae pre-treated with DMSO alone (Fig. 5). The MPL_250 μg/ml_-treated larvae showed a greater ability to migrate at high IVM concentrations compared to control larvae. This was demonstrated in two ways, first, the significantly higher IVM dose-response plateau for MPL pre-treated larvae compared to controls for WAL larvae at 3 h, and both isolates after 6 h pre-treatment (WAL 3 h 33.79 *vs* 17.60 %; Kirby 6 h 27.08 *vs* 4.27 %; WAL 6 h 37.05 *vs* 16.75 %) (Table 2); and secondly, the *t*-test analysis showing that the percent migration at the highest IVM concentrations was consistently greater (*P* < 0.05) than the percent migration at the same IVM concentration for controls (Fig. 5).

**Table 2:** Response of *Haemonchus contortus* larvae to ivermectin following pre-exposure to monepantel

Pre-exposure period	Isolate	Drug exposure	Ivermectin dose-response					
			IC_50_			Dose-response plateau		
			Drug conc.^a^ (μg/ml)	95 % CI	Drug/DMSO^a,b^	Migration^c^ (%)	95 % CI	Drug/ DMSO^c,d^
3 h	Kirby	DMSO	1.6	1.3–1.9	–	7.3	2.3–12.3	–
		MPL	2.5	1.6–3.8	1.5	18.3	7.5–29.2	2.5
	WAL	DMSO	4.0	3.5–4.6	–	17.6	12.7–22.5	–
		MPL	6.1	4.2–8.9	1.5	33.8*	25.8–41.8	1.9*
6 h	Kirby	DMSO	1.4	1.2–1.7	–	4.3	-0.06–8.6	–
		MPL	0.9	0.5–1.5	0.6	27.1*	18.1–36.1	6.3*
	WAL	DMSO	2.8	2.4–3.3	–	16.8	10.9–22.6	–
		MPL	2.5	1.1–5.6	1.03	37.1*	24.0–50.1	2.2*

^a^Within either the 3 or 6 h pre-exposure data sets, * denotes that the IC_50_ following pre-exposure to anthelmintic was significantly higher than the IC_50_ following pre-exposure to DMSO, as determined by non-overlap of 95 % confidence intervals

^b^Drug / DMSO = IC_50_ for IVM following pre-exposure to MPL/ IC_50_ for IVM following pre-exposure to DMSO

^c^Within either the 3 or 6 h pre-exposure data sets, * denotes that L3 migration at the dose-response plateau (%) following pre-exposure to MPL was significantly higher than L3 migration (%) following pre-exposure to DMSO, as determined by non-overlap of 95 % confidence intervals

^d^Drug / DMSO = L3 migration (%) for IVM following pre-exposure to MPL/ L3 migration (%) for IVM following pre-exposure to DMSO

In contrast to these changes in response to the highest IVM concentrations, the response of the remaining worm population did not change significantly, as shown by equivalent IC_50_ values, and a lack of statistical differences in percent migration at individual IVM concentrations (*t*-test analysis) (Table 2, Fig. 5). That is, the IVM tolerance was only observed in a component of the worm population. The only exception to this, in terms of the *t*-test analysis, was the decreased migration for MPL treated larvae at an IVM concentration of 0.2 μg/ml for WAL larvae (Fig. 5c). There were no significant changes in LEV IC_50_ values following exposure of Kirby and WAL larvae to MPL_250 μg/ml_ (see Additional file 2: Figure S1; Additional file 3: Table S2).

**Fig. 6:**
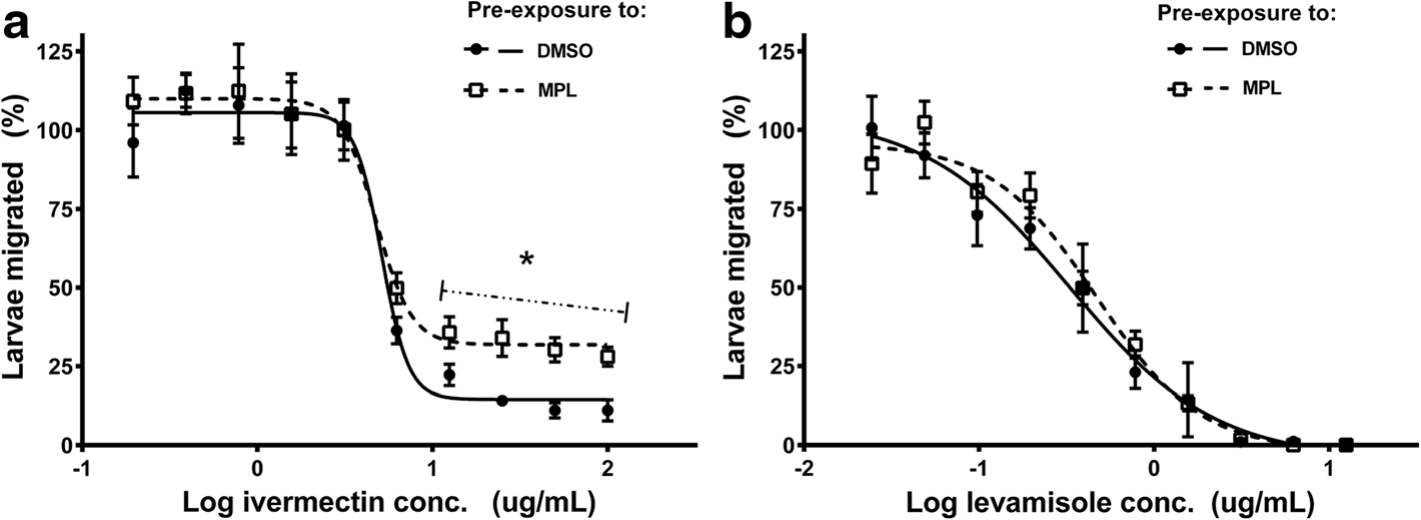
Stability of IVM tolerance in WAL L3 stage larvae pre-treated with monepantel. Sensitivity of WAL L3 stage larvae to IVM (**a**) or LEV (**b**) was measured using migration assays following pre-exposure for 3 h to MPL_250μg/ml_, followed by 24 h in water only, compared to L3 exposed to DMSO for 3 h, followed by water for 24 h. DMSO pre-exposure shown with solid lines and closed symbols, MPL pre-exposure shown with dashed lines and open symbols. The data points marked with * show significant differences (*P* < 0.05) in percent migration between MPL- and DMSO- pre-exposure groups (*t*-test). Each data point represents mean ± standard error of the mean (SEM), *n* = 9 (pooled data from three experiments, each with assays in triplicate)

WAL larvae that had been exposed to MPL_250 μg/ml_ for 3 h, then washed and held in water for a further 24 h, before being exposed to a range of IVM concentrations in LMAs, showed a similar pattern of IVM tolerance to that described above (Fig. 6). The IVM dose-response plateau was increased significantly (from 14.6 to 31.8 %), the *t-*test analysis showed significant differences at the four highest IVM concentrations, and there was no change in the IC_50_ (Fig. 6a). There were no significant changes in LEV IC_50_ values for these MPL-treated larvae compared to DMSO controls (Fig. 6b).

## Discussion

In this study, we have demonstrated that exposure of *H. contortus* L3 to a high concentration of MPL (250 μg/ml) results in increased transcription of a number of ABC-transporter genes. Furthermore, exposure to MPL at this concentration results in greater efflux of R-123 from the L3, as well as increased tolerance to IVM in a proportion of the larval population. In contrast, pre-exposure to a 100-fold lower concentration of the compound (2.5 μg/ml) only resulted in a single instance of low-level upregulation of a transporter gene (*pgp-11*), an increase in R-123 efflux in only one out of four isolate/ time point instances, and did not result in any IVM tolerance in subsequent migration assays. Hence, the effects of MPL on gene transcription, R-123 efflux and subsequent IVM tolerance were dependent on the concentration of the compound to which the larvae were exposed. This study is the first, to our knowledge, to indicate an interaction between MPL and ABC transporters in nematodes. However, our data describe the effects of MPL on the regulation of ABC transporter gene expression rather than a direct effect of the drug on the transporter proteins themselves. The demonstration of functional consequences of the gene transcription increases, in terms of R-123 efflux and IVM tolerance, indicates that the transcription increases most likely result in increased transporter protein activity towards R-123 and IVM. Hence, the present study provides evidence of an interaction of MPL with the regulatory mechanism for ABC transporter gene expression, and subsequent protein synthesis, rather than evidence as to whether MPL itself is a substrate for the transporters.

As described above, the transporter gene upregulation/R-123 efflux / IVM tolerance relationships were only observed at the high MPL concentration. This concentration was well above the levels that an adult worm would encounter in the abomasum of a sheep, as measured by Lifschitz et al. [21] to be 2–4 μg/g of abomasal content 48 h after administration of the drug. On the other hand, the lower concentration of MPL used in the present study, which was selected based on it approximating this in vivo concentration range, resulted in only one instance of transporter gene upregulation, and did not result in any IVM tolerance. It is clear that while the present study indicates an interaction between MPL at 250 μg/ml and the regulatory mechanism for ABC transporter transcription in *H. contortus*, it does not provide evidence for such a role in vivo. Further experiments would be needed to determine if MPL interacts with transporters in adult worms in vivo.

While the patterns of upregulation in Kirby and WAL L3s were quite similar at 6 and 24 h time points, the WAL larvae showed a much greater level of upregulation (in terms of fold increases as well as the number of transporters affected) at the 3 h time point compared to Kirby (compare Figs. 2d and 1d). Our earlier study, reported the upregulation of ABC transporters in the WAL isolate after exposure to IVM and LEV, alongside no changes in Kirby at the 3 and 6 h time points examined in that study [13]. This suggested that the rapid upregulation in response to these two drugs in the WAL isolate may be a component of the resistance shown by this isolate to the two anthelmintics. WAL and Kirby are both susceptible to MPL [20], having been isolated from the field before the use of this drug. The present study therefore indicates that this increased responsiveness of WAL larvae also occurs with respect to its response to drugs to which it is not resistant, and to which it has never been exposed. Thus, if the rapid upregulation following exposure to IVM and LEV is a component of the resistance shown towards these drugs as suggested by Raza et al. [13], then the present study suggests that this increased responsiveness may be quite general in nature, extending to xenobiotics beyond the anthelmintics that were involved in the original resistance-selection process in the field. Importantly though, while WAL larvae respond more rapidly and to a greater extent than Kirby to MPL under our experimental conditions, the two isolates show equivalent sensitivities to the drug in larval development assays [20]. Hence, there is no evidence yet that the increased responsiveness in ABC transporters seen in WAL in the present study provides any subsequent protection against MPL; only protection against IVM has been suggested by our data.

The pattern of transcription changes over the time course of the present experiments was quite different to that observed in our earlier experiments with IVM and LEV [13]. In this earlier study, exposure to either of the two anthelmintics resulted in ABC transporter transcription increases in WAL larvae at the 3 h time point, however, these had returned to control levels either completely (for IVM) or almost completely (with the exception of one gene for LEV) by 6 h. In contrast, in the present study, the upregulation response was sustained over the 24 h time period of the experiments. This difference between the two studies is most likely due to the relative concentrations of the drugs used. The concentrations were chosen on the basis of being the highest concentrations that could be examined without effecting the ability of the larvae to migrate in LMAs in the second phase of the drug-exposure experiments, thereby allowing for the consequences of drug exposure to be assessed using LMAs. As MPL is far less potent as an inhibitor of larval migration than IVM and LEV [20, 22], the higher of the two concentrations used in the present study was much higher than those used previously by Raza et al. [13] for IVM and LEV (250 μg/ml compared to 0.2–0.8 μg/ml).

It has been suggested that nematodes may use gene upregulation as a mechanism to counter the toxic effects of anthelmintics [23]. There is considerable evidence that anthelmintics act as substrates of ABC transporters and have inducing effects on the expression levels of transporter genes in nematodes and mammals [11, 24, 25]. However, nematode transporter genes do not show a consistent pattern of upregulation following exposure to anthelmintics. Significantly increased transcription of several P-gp genes was reported in wild-type and IVM-resistant isolates of *Caenorhabditis elegans* post-exposure to moxidectin [14]. On the other hand, exposure of the same isolates of *C. elegans* to IVM resulted in upregulation of a different set of P-gp genes [8]. Some studies on parasitic nematodes have reported increases in transcription of transporter genes following in vitro and in vivo exposure to IVM [12, 16, 26], whereas no transcriptional changes were observed in IVM-resistant *H. contortus* and *Cooperia oncophora* worms collected from animals treated with IVM compared to the worms collected from untreated control animals [27, 28].

We also examined the stability of the observed transcriptional changes by exposing WAL L3 to MPL_250 μg/ml_ for 3 h, rinsing the larvae in water, and then maintaining them in the absence of drug for a further 24 h. We found that only the transcription level of *pgp-11*remained at significantly higher levels compared to control larvae. We also observed increased tolerance to IVM in a proportion of the WAL larval population 24 h after the removal of MPL following a 3 h pre-treatment period (see Fig. 6). There is very little information available on the time course of ABC transporter gene expression patterns after the removal of the inducing agent. Fardel et al. [29] reported that expression of P-gp (*mdr-1*) in rat liver epithelial cells, as measured by qPCR and Western blotting, was significantly higher following exposure to 3-methylcholanthene for 24 h, with a return to almost basal levels 72 h after removal of the inducing agent. The sustained upregulation of *pgp-11* alone after removal of the MPL in the present study suggests that this specific ABC transporter may play a more important role in interaction with MPL in WAL larvae than the other transporters. On the other hand, the maintenance of IVM tolerance 24 h after removal of the MPL cannot be linked specifically to the sustained upregulation of *pgp-11* as the tolerance may be due to increased efflux activity of other ABC transporter proteins that may be present at higher levels as a result of the earlier period of gene upregulation (from Fig. 2d).

The presence of plateaus in IVM dose-response curves at the highest IVM concentrations was most likely due to the short nature of both the drug incubation and migration phases (30 min for each) of the LMA compared to the 48 and 24 h incubation/migration periods usually used for this assay [22]. As described earlier, the assay was modified to avoid any effects of the drugs in possibly inducing transcription of the transporter genes during long (24 and 48 h) incubation periods. These plateaus were likely due to the subsequent inability of IVM to inhibit migration completely in all larvae in the short 30 min time frame, and were also reported in our earlier study [13]. Tolerance to IVM only occurred in the proportion of the population represented by the dose-response plateau, as the IC_50_ in the remainder of the population was unchanged compared to DMSO pre-treated L3 (see Table 2). A proportion of the larvae were clearly better equipped to tolerate IVM following MPL_250 μg/ml_ exposure (as indicated by percent migration at plateau and *t*-test analysis), while the remainder of the population showed no change in IVM sensitivity. Although we have no direct evidence for a role of transporters in the observed IVM tolerance, the known role for ABC transporters in IVM tolerance [9] in parallel with our gene upregulation/R-123 efflux increases/IVM tolerance observations in the present study, suggest that the MPL-induced transporters were able to subsequently provide protection against IVM in a proportion of the larval population in our experiments. Hence, our data demonstrate the potential role of the transporters in responding to exposure to an anthelmintic, and, in so doing, provide protection against structurally-unrelated compounds. The fact that IVM tolerance was only observed in a proportion of the worm population following MPL pre-exposure may indicate a degree of heterogeneity in the effect of MPL exposure on ABC transporter gene expression across the worm population, as was also suggested by the similar IVM dose-response plateau versus IC_50_ effects following pre-exposure to IVM or LEV in our previous study [13].

There were no significant changes in LEV dose-response experiments following exposure to MPL at both concentrations, suggesting that the transporters induced in response to MPL exposure are able to subsequently provide protection against IVM but not LEV. Raza et al. [13] previously showed that pre-exposure to IVM or LEV resulted in tolerance to IVM, alongside no change in the response to LEV. Hence, the earlier and present studies provide evidence to suggest that IVM may be a better substrate than LEV for *H. contortus* ABC transporters, as has been reported for mammalian P-gps [30].

## Conclusions

In conclusion, the present study has shown that exposure to a high concentration of MPL increases the transcription of multiple ABC transporter genes in two MPL-susceptible isolates of *H. contortus*. The most significant interactions were for *pgp-11*, *pgp-12* and *pgp-14*, based on (i) the magnitude of the transcriptional response, (ii) the occurrence of this response across multiple time points, (iii) the consistency of the response between the two isolates, and (iv) the stability of the response upon removal of the MPL (applicable to *pgp-11* only). The subsequent ability of a proportion of MPL-exposed L3 to tolerate higher levels of IVM provides further evidence that ABC transporters play an important role in protection of worms against this anthelmintic, and illustrates the ability of ABC transporters to interact with different chemical entities. However, while the study describes interactions of ABC transporters with a high concentration of MPL in vitro, the effects of MPL on efflux pathways in vivo remains to be determined.

## Additional files

Additional file 1: Primer sequences used for the quantitative PCR used in this study. (DOCX 16 kb) Additional file 2: Levamisole dose-response curves following pre-exposure of the larvae to monepantel. (DOCX 179 kb) Additional file 3: Levamisole dose-response data following pre-exposure of the larvae to monepantel. (DOCX 13 kb)
