# Supplementary material for: Increased expression of ATP binding cassette transporter genes following exposure of Haemonchus contortus larvae to a high concentration of monepantel in vitro
Source: Parasit Vectors. 2016 Sep 29;9:522. doi: 10.1186/s13071-016-1806-9 (PMC5041279; doi:10.1186/s13071-016-1806-9)
Supplement: Additional file 1: — Primer sequences used for the quantitative PCR used in this study. (DOCX 16 kb) [file 13071_2016_1806_MOESM1_ESM.docx]

| No. | Primer | Sequences | Product size | Amplification Efficiency % | | | Source |
| --- | --- | --- | --- | --- | --- | --- | --- |
|  | Housekeeping genes | | | | | | |
| 1 | *Hc* GAPDH | F:TGGGTGTGAACCACGAGAC  R:GCAGCACCACGTCCATCA | 213 | | 91.17 | Sarai et al. (2013) | |
| 2 | *Hc* Actin | F:GAGTCATGGTTGGTATGGGAC  R:GGAGCTTCGGTCAAAAGTACG | 140 | | 88.80 | Sarai et al. (2013) | |
| 3 | *Hc* β-Tubulin | F: GCTTCCGCACTTTGAAACTC  R: TGAAGACGAGGGAATGGAAC | 160 | | 88.41 | This study | |
|  | ABC transporter genes | | | | | | |
| 4 | P-gp 1 | F:CCACATGCGCCACACCTTTTAG  R:AGACGACTCCGACGTAGTTTCG | 145 | | 84.67 | Sarai et al. (2013) | |
| 5 | P-gp 2 | F: GGACAAAAGCAGCGAATTGCC  R:ACAGACGATGCGCTACAATGAC | 169 | | 95.73 | Sarai et al. (2013) | |
| 6 | P-gp 3 | F: CCGGCAACTTGTACTTCAAGGC  R: TCACTGTGCTCTTTCCGCAAC | 94 | | 88.02 | Sarai et al. (2013) | |
| 7 | P-gp 9.1 | F: TCGACGGGAATCAAGAAATC  R: GCCCATCATTACGGAGAAGA | 168 | | 79.72 | This study | |
| 8 | P-gp 9.2 | F: CCAGTCCACCTCAATTCCAC  R: AACCGCTCACGTCTCTCTG | 65 | | 93.82 | Issouf et al. (2014) | |
| 9 | P-gp 9.3 | F: AGAAACAACGAATCGCCATC  R: TGTGCCACAACGATACAGGT | 157 | | 82.54 | This study | |
| 10 | P-gp 10 | F:TCAGAAAGATTATGCGCCACGG  R:CAGCGTCGAAGAGTCGGTAATG | 98 | | 88.94 | Sarai et al. (2013) | |
| 11 | P-gp 11 | F: ACCACGAAGCTGAACGAGAA  R: CACCAGAGTGATACGCCAGTC | 150 | | 93.20 | This study | |
| 12 | P-gp 12 | F: TGAGTTAGCCAACGCCAAAGG  R: ATGGCGATACGCTGCTTCTG | 111 | | 93.88 | Sarai et al. (2013) | |
| 13 | P-gp 14 | F: GCACTTGTCGGACCATCTG  R: GCTCTTGTCCAACTAATGC | 160 | | 89.60 | Sarai et al. (2013) | |
| 14 | P-gp 16 | F: AAAAGCGAGACAAGGTCGAA  R: TGTTTGGTTACCATGCTTGC | 165 | | 99.03 | Issouf et al. (2014) | |
| 15 | MRP 1 | F: GCCCGATTTCTTCGTTACTTC  R: TTTCTCAAGGGGTGCTGT-C | 125 | | 90.12 | This study | |
| 16 | MRP 5 | F: TGTCGGTAGAACGGGAAGTG  R: GCAGGGTATGCAAAGGAATAGA | 125 | | 85.28 | This study | |
| 17 | HAF-6 | F: CAATCAAACCCAGAGCGATAA  R: CAACAGCGAGCTTGAAACAG | 250 | | 80.62 | This study | |
| 18 | ABCF-1 | F: AAGGTGTCCGGCCTTAAGAT  R: TCAGTATGGATGTCGCTTGC | 146 | | 83.92 | This study | |
| 19 | ABCF-2 | F: ACGTGTAGCCTTGGTTGGTC  R: TTCAAGTGGGAGCTCTTCG | 157 | | 92.00 | This study | |

**Table S-1.** Primer sequences of housekeeping and ABC transporter genes used for the quantitative PCR used in this study.

**References:**

Sarai RS, Kopp SR, Coleman GT, Kotze AC: Acetylcholine receptor subunit and P-glycoprotein transcription patterns in levamisole-susceptible and -resistant *Haemonchus contortus*. Int J Parasitol Drugs Drug Resist. 2013; 3, 51-58.

Issouf M, Guegnard F, Koch C, Le Vern Y, Blanchard-Letort A, Che H, Beech RN, Kerboeuf D, Neveu C: *Haemonchus contortus* P-glycoproteins interact with host eosinophil granules: a novel insight into the role of ABC transporters in host-parasite interaction. PloS One, 2014; 9, e87802.
