# Supplementary material for: Increased expression of ATP binding cassette transporter genes following exposure of Haemonchus contortus larvae to a high concentration of monepantel in vitro
Source: Parasit Vectors. 2016 Sep 29;9:522. doi: 10.1186/s13071-016-1806-9 (PMC5041279; doi:10.1186/s13071-016-1806-9)
Supplement: Additional file 2: — Levamisole dose-response curves following pre-exposure of the larvae to monepantel. (DOCX 179 kb) [file 13071_2016_1806_MOESM2_ESM.docx]

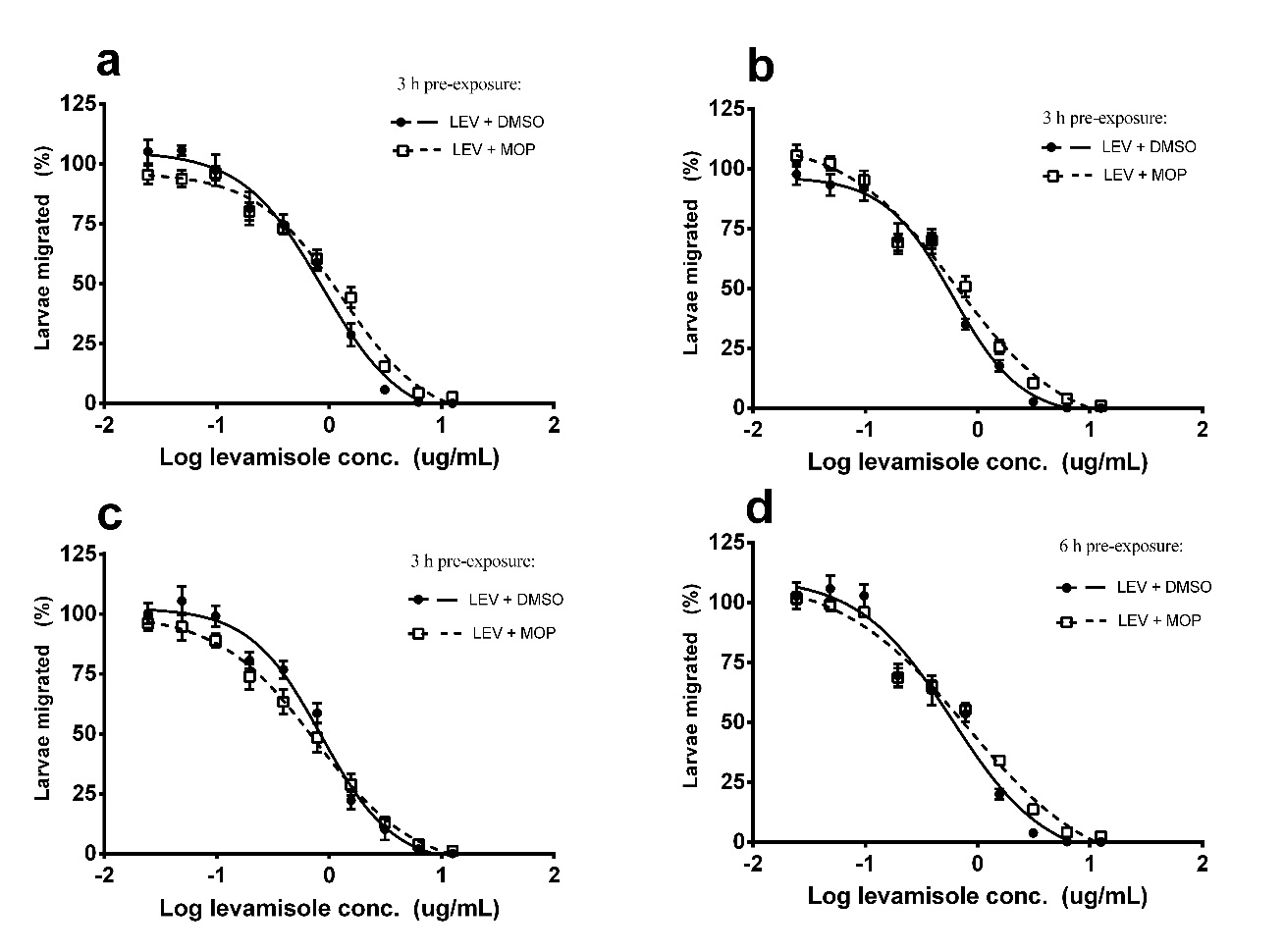


**Fig. S-1** **Monepantel pre-exposure and LEV sensitivity in Kirby and WAL L3 stage larvae.** Effects of MPL_250µg/mL_ pre-exposure on tolerance of Kirby (**a** after 3 h pre-exposure and **b** after 6 h pre-exposure) and WAL (**c** at 3 h pre-exposure and **d** at 6 h pre-exposure) L3 stage larvae to LEV in larval migration assays, compared to L3 pre-exposed to DMSO only. DMSO pre-exposure shown with solid lines and closed symbols, MPL pre-exposure shown with dashed lines and open symbols. Each data point represents mean ± Standard error of mean (SEM), *n* = 9 (pooled data from three experiments, each with assays in triplicate).
