# Supplementary material for: Increased expression of ATP binding cassette transporter genes following exposure of Haemonchus contortus larvae to a high concentration of monepantel in vitro
Source: Parasit Vectors. 2016 Sep 29;9:522. doi: 10.1186/s13071-016-1806-9 (PMC5041279; doi:10.1186/s13071-016-1806-9)
Supplement: Additional file 3: — Levamisole dose-response data following pre-exposure of the larvae to monepantel. (DOCX 13 kb) [file 13071_2016_1806_MOESM3_ESM.docx]

**Table S-2.** Response of *Haemonchus contortus* larvae to levamisole following pre-exposure to monepantel.

| Isolate | Drug pre-exposure |  | Levamisole dose-response | | | | | | |
| --- | --- | --- | --- | --- | --- | --- | --- | --- | --- |
|  |  |  | 3 hours Pre-exposure | | |  | 6 hours Pre-exposure | | |
|  |  |  | IC_50_ | | |  | IC_50_ | | |
|  |  |  | Drug conc.^a^  (µg/mL) | 95% CI | Drug/ DMSO ^a,b^ |  | Drug conc. ^a^  (µg/mL) | 95% CI | Drug/ DMSO ^a,b^ |
| Kirby | DMSO |  | 0.83 | 0.73-0.96 | - |  | 0.52 | 0.46-0.60 | - |
|  | MPL |  | 0.99 | 0.87-1.10 | 1.2 |  | 0.68 | 0.6-0.78 | 1.3 |
| WAL | DMSO |  | 0.82 | 0.72-0.94 | - |  | 0.63 | 0.54-0.73 | - |
|  | MPL |  | 0.64 | 0.56-0.75 | 0.8 |  | 0.74 | 0.66-0.83 | 1.2 |

**^a^** Within either the 3 or 6 h pre-exposure data sets, * denotes that the IC_50_ following pre-exposure to anthelmintic was significantly higher than the IC_50_ following pre -exposure to DMSO, as determined by non-overlap of 95 % Confidence Intervals.

**^b^** Drug / DMSO = IC_50_ for LEV following pre -exposure to MPL / IC_50_ for LEV following pre exposure to DMSO
